# Nonuse of Blended Web-Based and Face-To-Face Cognitive Behavioral Therapy for Alcohol Use Disorder: Qualitative Study

**DOI:** 10.2196/45471

**Published:** 2024-02-13

**Authors:** Kristine Tarp, Regina Christiansen, Randi Bilberg, Simone Borkner, Caroline Dalsgaard, Marie Paldam Folker, Anette Søgaard Nielsen

**Affiliations:** 1 Research Unit of Digital Psychiatry Centre for Digital Psychiatry Mental Health Services in the Region of Southern Denmark Odense Denmark; 2 Research Unit of Digital Psychiatry Department of Clinical Research University of Southern Denmark Odense Denmark; 3 The National Research Centre for the Working Environment Copenhagen Denmark; 4 Unit of Clinical Alcohol Research Department of Clinical Research University of Southern Denmark Odense Denmark; 5 Psychiatric University Hospital – University Function Mental Health Services in the Region of Southern Denmark Odense Denmark; 6 Department for Finance and Planning Lillebaelt Hospital University Hospital of Southern Denmark Vejle Denmark; 7 Brain Research Inter-Disciplinary Guided Excellence (BRIDGE) Department of Clinical Research University of Southern Denmark Odense Denmark; 8 Open Patient data Explorative Network (OPEN) Odense University Hospital Odense Denmark

**Keywords:** alcohol use disorder, blended internet-based and face-to-face cognitive behavioral therapy, nonuse, patient perceptions, qualitative

## Abstract

**Background:**

The use of digital technologies for health care has been the focus of social studies, which have concentrated on the digital divide between individuals who use technology and those who do not—with the latter often being considered as individuals with shortcomings. In Denmark, 91% of the population have computers and 97 out of 100 families have internet access, indicating that lack of access to technology is not the primary reason for nonuse. Although previous studies have primarily focused on participants' perspectives of using internet-based treatment for alcohol use disorder (AUD), no study has investigated individuals’ reasons to prefer face-to-face treatment over blended face-to-face and internet-based cognitive behavioral therapy (bCBT) for AUD among treatment-seeking populations.

**Objective:**

The aim of this qualitative study was to investigate the nonuse of bCBT among patients with AUD. Specifically, this study aims to explore patients' reasons for choosing not to receive treatment via this format.

**Methods:**

This study was conducted among Danish patients with AUD who were enrolled in the study “Blending internet treatment into conventional face-to-face treatment for alcohol use disorder (Blend-A)” but had not used bCBT. The participant group consisted of 11 patients with AUD: 3 women and 8 men. The age range of the participants was 29-78 years (mean 59 years). Individual semistructured interviews were conducted using cell phones to gather participants’ reasons for not choosing bCBT. The interviews were recorded, transcribed, and analyzed using thematic analysis. Five authors performed the analysis in 3 steps: (1) two authors read the transcripts and coded themes from their immediate impression of the material, (2) one author provided feedback, which was used to group overlapping themes together or create new themes that better reflected the content, and (3) the remaining two authors provided feedback on the analysis to improve its structure, readability, and relevance to the research aim.

**Results:**

We found that the participants had various reasons for choosing face-to-face treatment over bCBT; these reasons were more related to personal matters and lesser to digital health literacy. We identified 4 themes related to personal matters for choosing face-to-face treatment over bCBT: (1) patients’ need for attending sessions in person, (2) preference for verbal communication, (3) desire for immediate feedback, and (4) feeling more empowered and motivated with face-to-face sessions.

**Conclusions:**

This study provides valuable insight into participants’ perspectives on blended therapy for AUD and highlights the importance of considering personal factors when designing digital health interventions. Our study indicates that most of the participants choose not to use bCBT for AUD because they perceive such treatment formats as impersonal. Instead, they prefer direct communication with the therapist, including the ability to express and comprehend facial expressions and body language.

**International Registered Report Identifier (IRRID):**

RR2-10.1186/s12888-021-03122-4

## Introduction

### Background

Social studies of the use of digital technologies for health care have focused on the digital divide—the division between individuals using technology and those who do not—the latter viewed as merely individuals with deficits [1,2]. These deficits may cover a range of difficulties and barriers, which can occur when engaging with technology [3]. In general, social groups with higher education and higher income seem to have more knowledge, motivation, and competency in initiating steps toward a healthy lifestyle [4], in addition to having fewer barriers toward digital solutions [5]. For example, Heponiemi et al [6] described how individuals who do not use computers have lesser education, higher unemployment, lower income, and poorer health, and found a risk of digital exclusion among those who have lower socioeconomic status, poorer health, or are more socially isolated. Nonetheless, in Denmark in 2020, 97 of 100 families had internet access, 96% had a mobile phone, and 91% had a computer [7], which indicates that people in Denmark are regular users of digital technologies. Therefore, it may be anticipated that digital treatment interventions targeting individuals with alcohol use disorder (AUD), like guided internet-based cognitive behavioral therapy (iCBT), may be an appreciated intervention among the Danish population, although there may be barriers toward seeking treatments for AUD in general and specifically toward digital solutions.

A review [8] has shown the general barriers toward seeking traditional AUD treatments. For example, Wallhed Finn et al [9] conducted a study among nontreatment seekers with alcohol dependence on their perceptions of alcohol consumption, dependence, and barriers toward seeking face-to-face (FtF) treatment for AUD. They found participants to be generally negative toward FtF treatment, for example, due to stigma and shame. For this group of nontreatment seekers, an internet-based intervention like iCBT can be perceived as a potential first step toward entering treatment—both to assess one’s alcohol use and to receive guidance for suitable treatment.

Barriers toward engaging in iCBT for AUD have not been investigated much. In a study on attrition during a web-based treatment for problem drinkers, Postel et al [10] found the second most common reason for noncompletion of an internet-based intervention to be dissatisfaction with the intervention itself, for example, that it was too time consuming or demanding and did not meet personal needs. In another study of user experiences of internet-based treatment for problematic alcohol use, Ekström and Johansson [11] identified the following barriers toward engaging in internet-based AUD treatment: lack of recognition in the content of the intervention, too much text and repetition, too little (meaningful) support or feedback, lack of contact with a therapist, and lack of guidance.

Combining iCBT with FtF CBT is referred to as blended CBT (bCBT). bCBT for AUD may propose a treatment solution that combines a high level of discretion and flexibility in addition to being guided and person-centered [12]. It might, so to speak, offer the best from both the aforementioned treatment modes. Participants in that study [12] were offered bCBT, but they opted out of using bCBT as they preferred solely FtF CBT. We found this intriguing and important since we anticipated that bCBT, in particular, would be perceived as an attractive offer due to the high familiarity of the Danish population with digital technology. Thus, in this study, we wished to explore participants’ reasons for deciding against and opting out of using bCBT. To our knowledge, no study has previously investigated patients’ reasons to prefer FtF over bCBT or iCBT for AUD among treatment-seeking populations.

### Aim

In this study, we sought to understand individuals’ perceptions of bCBT and iCBT for AUD when they are introduced to this type of treatment format by the therapists. In particular, we aimed to explore participants’ reasons for choosing a treatment strategy that solely consists of FtF treatment and not digital solutions, when offered the possibility of a flexible combination of FtF and iCBT.

## Methods

### Settings

This study is a substudy under the study “Blending internet treatment into conventional face-to-face treatment for AUD (Blend-A)” [12]. At the beginning of the overall study, 18 Danish municipal treatment institutions participated, but only 14 clinics remained throughout the whole study period. The clinics are quite similar in structure and treatment content offers. In Denmark, the municipalities offer AUD treatment free of charge to the individual patient. The treatment is based on treatment manuals stemming from evidence-based treatment methods such as CBT and motivational interviewing (MI) [13]. A typical treatment course entails acute treatment for withdrawal symptoms, followed by a series of either individual or group-based sessions. The duration of the treatment courses depends on the patients’ needs.

A treatment layout for AUD that consists of a combination of FtF treatment and internet-based modules, which was developed in The Netherlands [14], was translated and adjusted to fit Danish language and culture [15]. During the Blend-A study, all patients who entered AUD treatment in the participating clinics were offered to receive all or part of their treatment course in Blend-A. Blend-A is operated as bCBT, where patients can use iCBT on a web-based platform, hosted by the Dutch company Minddistrict, in combination with attending FtF CBT sessions at the clinic. The degree of blending is agreed upon among specific clinics, therapists, and patients. One example is that, when blending, the patient would attend FtF CBT every sixth instead of fourth week. The platform entails 21 sessions with written material, visual resources, and assignments following CBT and MI. The therapist can offer a short paragraph of written feedback on some of the solved assignments for further elaboration during the FtF CBT. The format is flexible and the patients can access the web-based platform when it suits them. The platform can be accessed anonymously, if needed. The patient can go back and look at the earlier solved assignments, if needed. The implementation of the study commenced in June 2020 and ran until ultimo December 2022.

### Participant Recruitment

Participants in this study were recruited among the participants in the Blend-A study who did not engage in bCBT. In total, 1033 participants were enrolled in the Blend-A study; of these, 606 (58.6%) did not register for an iCBT profile on the web-based platform, and thus, did not make use of the bCBT offer. All Blend-A participants filled out a baseline survey and were invited to fill out a 6-month follow-up survey, no matter to which degree they had made use of bCBT for AUD, if at all. The 6-month follow-up survey was collected electronically or on the telephone by researchers, not knowing until the last questionnaire, whether the Blend-A participants had actually used bCBT for AUD. A random sample of 60 participants participating in the Blend-A study without bCBT were telephoned by author SB and invited to participate in telephone-based individual interviews for this study about their reasons for not wanting to use bCBT for AUD. Some did not answer the telephone, some did not feel that they could contribute as they could not remember having been offered bCBT, and some did not wish to participate and gave no reason for this. Twelve participants agreed to participate and scheduled an appointment for an interview; in 1 case, however, we failed to reach the participant.

### Data Collection

Data were collected using semistructured individual interviews with an interview guide, available in Multimedia Appendix 1. The interview guide was not pilot tested, and no repeat interviews were performed. The questions were inspired by relevant subjects found in the literature, asking about the participants’ background, experiences with using digital technology in their everyday lives, and their reasons for not choosing the offered bCBT for AUD. Furthermore, the questions were open-ended, leaving room for pursuing any given direction set by the participant. The interviews were conducted by a psychology student intern, Jakob Godsk Nielsen, and the first author KT. Neither were involved in the clinical treatment in this study. No relationship between the interviewer and interviewee was established prior to study commencement. The interviewees had no prior knowledge about the researchers, and no characteristics about the interviewers were reported to the interviewees other than that the interviewers were researchers. The interviews were conducted over telephone, lasted between 30 minutes and 45 minutes, and were audiotaped and transcribed in NVivo (QSR International) in full length by authors SB and CD. The transcribed interviews were not returned to the interviewees for commenting and corrections, and no field notes were made during the interviews. We used COREQ (Consolidated Criteria for Reporting Qualitative Studies) [16] as a checklist for reporting on the interviews. Data were anonymized and securely stored.

### Data Analysis

The transcribed interviews were analyzed in the qualitative software support system NVivo by using thematic analysis [17]. First, all transcripts were read to obtain an overall immediate impression of the material. Along reading, the material was coded by themes that came to the mind of the authors (KT and RC, both female postdocs, who holds MA degrees in anthropology and philosophy, PhD degrees within health sciences, and approximately 10 years of experience within the field). Second, another author (ASN) commented on the transcripts with the coded themes. Based on these comments, the authors (KT and RC) recoded the material with focus on overlapping themes grouped together or recoded with new themes that more accurately specified the content. Lastly, the 2 remaining authors (RB and MPF) gave their feedback on the themes, structure, and readability of the analysis, leading to the final themes as expressed by the participants who had chosen not to use bCBT. The participants did not provide feedback on the findings. The collected themes are further described in the forthcoming results section.

### Ethics Approval

This study was conducted according to the current ethical standards. The protocol for the Blend-A study was approved by the scientific research ethics committee of the Region of Southern Denmark (project identification S-20190166G). The Danish Data Protection Agency gave the permission to collect and store data (record 20/12692). After receiving both oral and written information about the study, the participants signed a consent form. Further, participants were informed about their rights to withdraw their consent at any time, without any consequences on their treatment course.

## Results

### Participant Sample Description

Ultimately, 11 participants participated in this nonuse study. The baseline characteristics of the participants are shown in Table 1. The participant group consisted of 3 women and 8 men, with a mean age of 59 (SD 16) years. The youngest participant was 29 years, and the oldest participant was 78 years. Five of the participants were married or in a relationship, 5 were single, and 1 was widowed. The participants were all educated—either within craftsmanship or had a short, intermediate, or long higher education. Five were currently employed, 3 were unemployed, and 3 had retired. They all described having an everyday schedule, wherein they got up and went about their daily activities. Besides, 2 of them had additional mental illness, and 4 had somatic illness. The participants had used alcohol problematically for 3-30 years (mean 11 years). Their reasons for seeking treatment were to find someone to talk to and to receive help, support, and advice. They expressed that they needed tools to reduce their alcohol use. During the interviews, the participants self-assessed themselves to be super users of technology (n=2), intermediate users (n=7), and having limited digital competencies (n=2). Compared to the profile of 44,516 patients, who had a total of 88.057 treatment courses in Danish alcohol treatment institutions between 2006 and 2014 [18], our study sample was somewhat older.

**Table 1:** Baseline characteristics of our study participants compared to those of patients seeking treatment in Danish alcohol treatment institutions in 2006-2014.

	Nonuse sample in this study (N=11)	National Danish profile [18]
Age (years), mean (SD), range	59 (16), 29-78	46-49^a^
Excessive alcohol use (years), mean (SD), range	11.19 (7.41), 3-30	12-13^a^
Sex (female), n (%)	3 (27)	30-31^a^
Married/in a relationship (yes), n (%)	5 (45)	41^b^
Education (low), n (%)	6 (55)	49^b^
Education (intermediate), n (%)	2 (18)	16^b^
Education (high), n (%)	3 (27)	8^b^
Employment (yes), n (%)	5 (45)	38^b^
Additional mental illness (yes), n (%)	2 (18)	4^b^
Somatic illness (yes), n (%)	4 (36)	N/A^c^
Technology user (low level), n (%)	2 (18)	N/A
Technology user (intermediate level), n (%)	7 (63)	N/A
Technology user (high level), n (%)	2 (18)	N/A

^a^These data represent ranges in percentages as mentioned in [18].

^b^Values are presented in percentage, as the exact n values are not provided in [18] and cannot be calculated.

^c^N/A: not applicable.

### Description of Themes

Two participants considered themselves to be technology super users, 7 felt that they were intermediate users, and 2 felt that they were inadequate digital users. The 2 latter participants felt that they had insufficient digital competencies for receiving treatment via the internet, as they did not understand it and felt unacquainted and terrible at it. One participant elaborated as follows:

...I am really bad at internet and all such technical stuff. I am also old. I did not grow up with it. But during that time where I had to be able to use it at work, I learned the basics to manage. Besides that, I have never done more about it. And when I stopped working, I also got rid of the internet. I simply don’t use it.

Participant ID 1002

It was unclear if the participant was reluctant about bCBT because of insufficient digital competencies or if the participant merely did not find technology use engrossing. The participant did not believe that he or she had the digital competencies to make use of bCBT, even though the participant had been a former internet user and had used technologies at work. However, in general, neither this nor the other participants reported feeling insecure or unsafe about using the internet as such. Nine out of 11 participants who chose to receive FtF CBT instead of bCBT for AUD explained that their choice had rather to do with them feeling a need for attending the sessions in person. This need consisted of multiple facets. The participants seemingly felt being exposed by having to enter a treatment center, thereby accepting that someone might recognize them. They described that when having reached this far in surrendering to the fact that they had an alcohol problem, they could not risk that treatment could fail. The participants had a wish to gain as much as possible from the treatment course, considering FtF CBT to be the safe route to success.

The participants considered that when feelings were involved, there was a risk that they would become emotionally upset during the process. When being upset, they anticipated that there might be a difference between being at the computer alone and being in a room with a person who might tell if you were, for example, anxious. One participant described how being in a vulnerable position demands a level of maintenance, which may succeed through verbal communication upheld by the therapist, as this could offer dialogue, nuance, and reflection, asking more deeply into and seeing behind difficult issues. The participants seemed to link physical appearance with the ability to move the therapy forward. Some of the participants explained that videoconferencing and telephone calls would also be okay if it was a synchronous conversation but not as a substitute for physical attendance during treatment.

Accordingly, an often mentioned factor that influenced the participants’ choice was that they considered internet-based treatment to be impersonal and that they preferred to be physically present in the same room as the therapist. The perceived benefit of FtF CBT was, according to the participants, the possibility of instantaneous communication. The participants believed being physically near to the therapist would enable a more trustful relationship, as described below:

…here people in question need help, and they need a pat on the back when things go well and help when things go bad. I do not think you can do that over mail (… with an email, you just become a number in a line, instead of a person who needs a shoulder to cry on and an ear that minds to listen.…..) When the matter is alcohol, then I do not think it is something that can take place over an email. Because then I think that I would feel pissed on.

Participant ID 957

The above quote shows how participants perceive internet-delivered therapy to be impersonal—a concept often used in digital technologies although not specified. The quote above shows that what constitutes not perceiving the FtF treatment as impersonal is the ability to feel the presence of the therapist and having the feeling of being understood and respected—something that the participant considered was difficult to be accomplished over an email from the therapist. A participant considered that to receive an email, even if it is a part of the internet-delivered treatment program, was equal to being as a number in a line.

Another factor that had an impact on the participants preferring attending sessions in person was the ability to see the therapists’ body language and look at them into their eyes during the sessions. This ability led the participants to believe that they could better comprehend the therapists and their responses. Being able to have questions elaborated on and clarified immediately was of importance to 5 of the participants. One example concerned personally sensitive subjects, where the participants found it easier to receive a response if they articulated the matters in a conversation compared to an email exchange, wherein the therapist might not have the time to answer right away. The participants’ wish was to have such issues settled instantly. One participant elaborated on the importance of receiving a quick clarification on outstanding matters:

…If I am in front of the therapist I am talking to, I would be able to get a response right here and right now. Then I can park it and it does not have to live inside my body anymore. Then it is out of the body. Away, fine, it is gone, finished (…) then I can move on. Then it doesn’t live inside my brain, fill up space, or spend resources anymore.

Participant ID 1091

The immediate feedback was of importance to the participants in situations where they felt alone and in doubt about how to understand a question. The physical presence of the therapist enabled them to receive a quick clarification and thus be able to move on. The verbal communication made sense to the participants as they felt safer and assessed it to be more giving. They felt that they could gain more from verbal communication because they could tell, explain, and inform, which enabled them to instantly see reactions or signals from the therapist, which they needed to act on, or give the therapist the possibility to ask questions allowing the participant to elaborate. It was their experience that a message is better understood when you look into each other’s eyes while communicating, as it is easier to reflect on what has been said and let it sink in before one answers and then give a more precise answer based on the discussions and the reflections based on the discussion. Below, is the transcript of one participant as a voice for all:

…It is because there is some communication that you cannot always see, and something happens when you talk to people that does not happen when you write. What happens is that you reflect differently when you have a conversation and a dialogue. It is also easy to write that “all is well.” In a person, you can see if it is and maybe say “well, are you sure about that?” (Laughs).

Participant ID 1128

Another advantage of FtF mentioned by the participants was that sitting in front of the therapist made them feel more obliged to adhere to the treatment or more compliant in relation to not drinking. The participants expressed how they felt dutiful and believed that if they had agreed upon attending an FtF CBT session, they would not cancel it. They imagined that it would be easier to cancel an internet-based session, thereby giving them an opportunity to choose the easy way out.

The participants also believed that it was beneficial that FtF therapy enabled synchronous sessions with the therapist, while on the web-based platform, the correspondence is asynchronous and the participants can use it when they want, which they believed to be risky for them and thereby the treatment. Sessions held in person make it more difficult to cheat the therapist with regard to drinking compared to web-based sessions, where they considered that it would be easier to continue their drinking. One participant unfolded this drawback as follows:

…When you make an agreement, I think it is nice that you can look each other into the eyes. Especially when it is about alcohol, then I cannot just say “I promise.” There is just something about the human contact.

Participant ID 957

In other words, the participant considered that the commitment is stronger if expressed FtF to the therapist compared to in writing during a web-based session. Thus, choosing solely FtF CBT rather than also making use of internet-based solutions meant deciding on taking responsibility for and committing strongly to their own treatment. Finally, the participants reported that FtF CBT sessions enable them to concentrate on their situation and focus on the treatment—a dimension that is perceived as necessary to maintain the consistency in their rehabilitation. Thus, we suggest that participants gauge their need for treatment and choose the treatment that best suits them and would be beneficial for them. Participants in this study are aware that they will not continue their treatment if it fails in their life and find that FtF CBT is a better option than just “keeping up appearances” through internet-delivered therapies.

## Discussion

This study aims to investigate the perceptions that are prevalent among participants who decide to opt out of the possibility of using bCBT for AUD and instead continue with FtF CBT without merging it with internet-based modules. We found that the participants had various reasons for preferring FtF CBT over bCBT, and these reasons were mainly related to personal choices.

### Participants’ Assumed Need for Attending Sessions In Person

Being physically in front of the therapist was considered to strengthen a more personal connection between the participant and the therapist and thus the central reason for preferring a synchronous FtF verbal dialogue. Participants considered that FtF allows for all aspects of communication with the therapist to come into play, including nonverbal communication, eye contact, and body language. In a systematic review on women’s expectations and experiences regarding eHealth treatment, Verhoeks et al [19] found 3 studies that showed women’s negative expectations with regard to receiving eHealth treatment. Those studies showed that the eHealth treatment was perceived as rather impersonal treatment and that the participants valued immediate and empathic responses in their dialogues with the therapist and stressed the importance of nonverbal communication through eye contact and bodily expressions. In the study by Verhoeks et al [19], the women expressed an intuitive preference for FtF CBT. They feared that the absence of personal contact would make their treatment course more impersonal and impact negatively on their alliance with the therapist, their motivation, and consequently on their treatment outcome.

In this study, we found that the participants had similar feelings as they took their rehabilitation process seriously. They chose the treatment form that they believed they could gain the most from—to them, it was FtF CBT. In general, we found that participants choose FtF CBT rather than bCBT because they had been in a vulnerable position in their rehabilitation process. In particular, it was of importance to them to have a sense of privacy and having a person in front of them when the subject is a personal and vulnerable matter. Since some of the participants had recently stopped drinking, they felt vulnerable in the situation because they realized how alcohol had got hold of them and now were dependent on a therapist’s assistance and guidance to help reduce their alcohol use.

### Participants Preferring Verbal Communication

In particular, the participants in our study considered verbal communication to be better than digital written communication to express dialogue, nuances, and reflections and to allow clinicians to ask deeper questions and grasp difficult issues. Further, the participants considered that they would gain more from verbal communication than from digital communication, as they assumed that it enabled clinicians to immediately react to participant’s signals and ask questions about their understanding of the said. The review of Verhoeks et al [19] also reported difficulties in explaining complex situations and feelings in written text compared to communicating through verbal FtF sessions. In their study [19], participants commented that they were afraid that the therapist would misunderstand their issues given in writing. In this study, the participants stressed the importance of a conversation, in which they would be able to ask questions and discuss problems with the therapist because they needed a more in-depth dialogue about their problems. These findings are corroborated by Runz-Jørgensen et al [20]. In their study, the participants perceived web-based treatment as undesirable because the therapist would just be waiting for the next person in line and they felt neglected. It should, however, be noted that not all participants agreed with the above interpretation. Other studies report that participants have used text-based interventions for AUD and have found them to be a positive experience in their treatment course [21,22].

### The Meaning of Receiving Immediate Feedback on Outstanding Matters

We found that the participants emphasized a need for immediate feedback from their therapist on outstanding matters—a need that they felt that asynchronous digital communication could not fulfil. In situations where they felt alone and in doubt about how to understand a question, it was of importance for them to receive instant elaboration on the matter. Here, the physical presence of the therapist enabled them to receive a quick clarification and thus be able to move on in their rehabilitation. This finding is congruent with findings from the review by Verhoeks et al [19], where the women wished to be able to ask questions and receive feedback from their therapist during their treatment course. The women stressed the importance of physical presence as they otherwise doubted the quality of the feedback. In continuation of this, participants in the study by Runz-Jørgensen et al [20] had a wish for even longer FtF consultations with the therapist as they felt that there was not enough time to ask questions or address concerns that were of importance to them.

It can be hypothesized that integrating videoconference-based conversations with therapists in digital treatment solutions for AUD might acknowledge and apprehend participants’ preferences in terms of being able to communicate at a distance without the loss of sensorial stimulation. Further research is thus needed in order to secure that digital solutions become attractive and preferred by participants. We anticipate that our findings may be used for developing information material addressed to therapists regarding participants’ concerns toward bCBT for the therapist to accommodate the potential participant barriers beforehand. Moreover, our findings may be used to inform participants prior to treatment about their possibilities of combining treatment forms in accordance with their specific needs at specific times.

### Strengths and Limitations

This study has limitations. The relatively small sample size (N=11) may be a limitation in this study. However, in an experiment with data saturation and variability, Guest et al [23] found the first 6 interviews to be crucial for the emerging of meta themes. Based on this finding, they recommended a minimum of 6 interviews for developing meaningful themes during an inductive analysis. Further, Crouch and McKenzie [24] found that a small number of participants is usable for facilitating the interviewer-interviewee alliance, thereby increasing the validity of semistructured interviews. This study is strengthened by the use of independent parallel coding and code check, which increase internal validity and reliability [25] and thus enhance the credibility of the analysis [26,27]. However, it may be a limitation that we have not used stakeholder check [27]. We saw that, compared to the profiles of the patients in Danish alcohol treatment institutions, our participant population was older. This difference in sample population characteristics may have had an influence on our study results. The relatively higher mean age in our sample may have had an impact, as participants may have less experience with and thereby less interest in using digital interventions [28]. It is also a possibility that the subjects covered in the interview guide and the way of asking by the interviewers may have affected the answers given by the interviewees, but it does not change the fact that they initially did not make use of the bCBT offer.

### Conclusion

We found multiple reasons for participants choosing FtF CBT over bCBT. Participants expressed a preference for FtF, in particular, due to positive expectations in the various dimensions of FtF, which they felt were important. The participants were worried that they would not feel as motivated, empowered, and obliged to complete treatment if it partly consisted of iCBT, as they would if it purely consisted of FtF sessions with a therapist.

## Appendix

Multimedia Appendix 1 Interview guide for patients who opted out of using blended internet-based and face-to-face treatment for alcohol use disorder.

## Abbreviations

AUD: alcohol use disorder
bCBT: blended cognitive behavioral therapy
Blend-A: Blending internet treatment into conventional face-to-face treatment for alcohol use disorder
COREQ: Consolidated Criteria for Reporting Qualitative Studies
CBT: cognitive behavioral therapy
FtF: face-to-face
iCBT: internet-based cognitive behavioral therapy
MI: motivational interviewing

## Data Availability

The data sets on which this study is based will be made available by the authors on request.
