# Supplementary material for: Nonuse of Blended Web-Based and Face-To-Face Cognitive Behavioral Therapy for Alcohol Use Disorder: Qualitative Study
Source: JMIR Form Res. 2024 Feb 13;8:e45471. doi: 10.2196/45471 (PMC10900073; doi:10.2196/45471)
Supplement: Multimedia Appendix 1 [file formative_v8i1e45471_app1.docx]

**Multimedia Appendix 1. Interview guide for patients who opted out of using blended internet-based and face-to-face treatment for alcohol use disorder.**

|  | |
| --- | --- |
| **Introductory questions:** | *Thank you for participating in this interview. If you consent, I would like to record it. The interview will be about your reasons for opting out of using blended internet-based and face-to-face alcohol treatment. It is totally okay that you have chosen this way, we are just curious about the background for your choice. I will pose some introductory questions asking about you and your life in general. Then I will pose some questions more specifically asking about your reasons for opting out of blended alcohol treatment. I will finish of with some concluding questions. I will start asking questions, you are welcome to explain and describe in the direction the experiences and perceptions occur to you. At first, the introductory questions.*   - What is your name (and sex)? - May I ask about your age? If yes, what is your age? - What is your marital status? - What is your education? - What is your occupation? - Will you describe a normal every day for you? - Do you suffer from any diseases? If yes, which? - If you want to tell me, I would like to hear about for how many years you have been drinking. - How often do you use a computer or tablet in your every day? How do you rate your own user skills? - Do you feel safe using the internet? - Is there anything else or something more you would like to tell me about your background? |
| **Questions about opting out of blended alcohol treatment:** | *Then I will move on to the main questions, more specifically about your reasons for opting out of blended alcohol treatment.*   - What did you need when you approached the clinic (e.g., someone to talk to, getting tools, medicine or the like)? - What did your therapist tell you about the possibility of receiving some of your treatment course from home via the internet?   - How did you feel about this opportunity?   - What did you imagine blended treatment would be like? - How do you feel about being able to choose between internet-based, face-to-face, or blended treatment? - How do you feel that you are best able to understand other people’s messages? And how do you prefer to communicate with others (verbally/in writing)? What are the advantages of the preferred way and the disadvantages of the non-preferred way? - How do you find that you can better or more easily express yourself (verbally/in writing)? - What is the difference between receiving a mail from your therapist and come in person to the clinic?   - What do those two things give you?   - Something different? - Are there situations where you consider online treatment to be a good option?   - What would you choose if you should choose now? - Do you have anything else you would like to say related to your decision that blended treatment is not an option for you? |
| **Concluding questions:** | *Then I will finish off with the concluding questions.*   - Are there anything more you would like to say? - Do you have any questions you would like to ask?   *Thank you very much for participating in this interview and share your experiences. The interview will be anonymized og transcribed. Then the interviews will be analyzed and disseminated in an article.* |
